# Management of a rare case of anterior cruciate ligament reconstruction in a Paralympic athlete with a transtibial amputation – a case report

**DOI:** 10.1051/sicotj/2025022

**Published:** 2025-04-08

**Authors:** Alexandre Le Guen, Thibaut Lucena, Eric Laboute, Etienne Cavaignac

**Affiliations:** 1 Department of Orthopaedic Surgery and Trauma, Hôpital Pierre Paul Riquet Place du Dr Baylac – TSA 40031 31059 Toulouse cedex 9 France; 2 C.E.R.S., Groupe Ramsay Santé 83 Av. Mal de Lattre de Tassigny 40130 Capbreton France

**Keywords:** Amputee, Anterior cruciate ligament, Arthroscopy

## Abstract

Advances in technology, prosthetic components and rehabilitation techniques have improved the quality of life for amputees. Wearing a prosthesis enabled them to participate in sports at a high level. Participating in competitive sports puts them at risk of joint injury. This case describes a disabled professional paralympic athlete with a transtibial amputation who has torn his anterior cruciate ligament (ACL). This patient underwent anterior cruciate ligament reconstruction one year before the Paris 2024 Paralympic Games. Surgery had to be adapted in terms of the patient’s operative position, choice of graft and incisions to limit conflict with the prosthesis. Anterior cruciate ligament reconstruction with an ipsilateral quadriceps tendon graft enabled the patient to return to competition and place 4th in his category at the Paris 2024 Paralympic Games. This is the first case of ACL reconstruction in a transtibial amputee reported in the literature. It highlights a rare and difficult surgical procedure that can yield good results.

## Introduction

Advances in technology, prosthetic components and rehabilitation techniques have improved the quality of life for amputees who wear a prosthesis. These custom-made prostheses are highly individualized for each patient and allow them to participate in adapted sports. Although this has never been described in scientific literature, elite athletes with below-knee amputation can suffer ligament tears in the knee. This work describes an anterior cruciate ligament (ACL) reconstruction in a 34-year-old male Paralympian medalist in the long jump at the Tokyo 2021 Paralympics. In this context, the ligament reconstruction procedure required specific adaptations in terms of graft selection and incisions to avoid pressure points on the prosthetic socket.

## Case report

**Figure 1:**
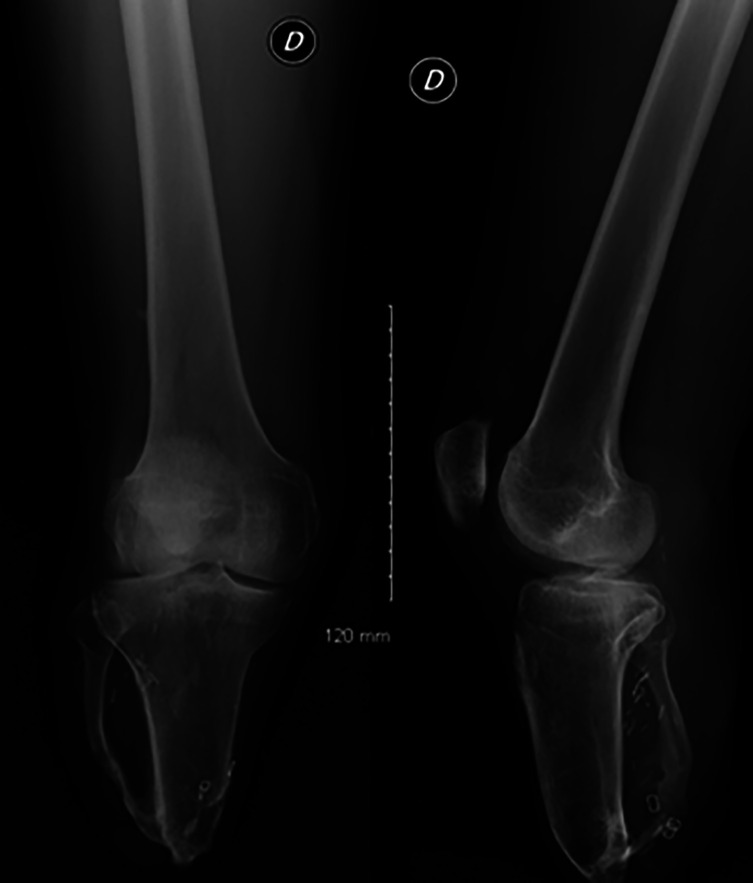
X-ray of the right knee showing the amputation level with a stump length of 12 cm.

This case involves a 34-year-old man who underwent a right transtibial amputation performed 12 cm below the joint line in 2007 following a work-related injury (Figure 1). He was a member of the French Paralympic team in athletics and a medalist during the 2021 Paralympics.

**Figure 2:**
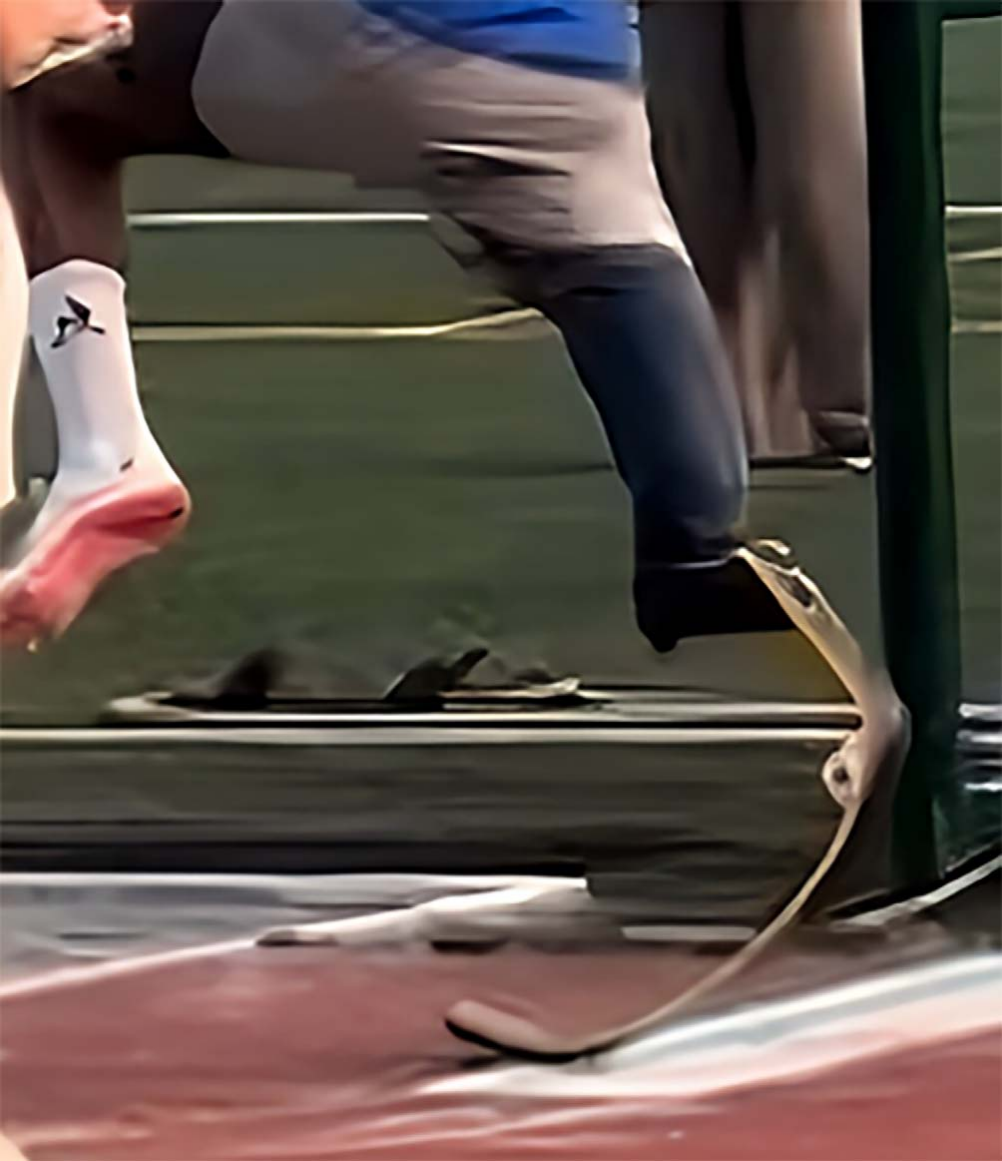
Right Knee hyperextension injury.

In July 2023, he suffered a hyperextension injury of the knee on the amputated side while training for the long jump (Figure 2).

**Figure 3:**
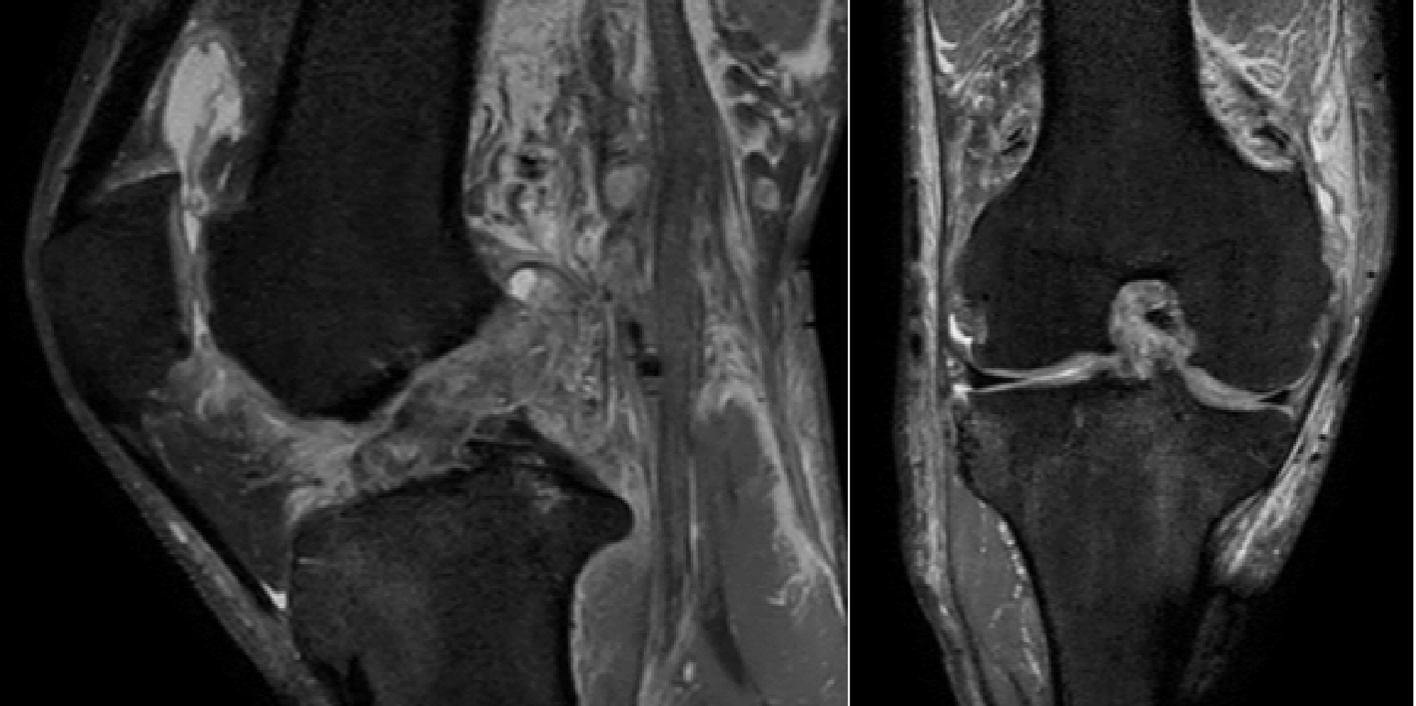
MRI image showing the ruptured ACL*.*

Clinical examination 7 days after the injury revealed mild knee joint effusion (stroke test), a Lachman test with no firm endpoint, no rotational instability and no coronal plane laxity in 0- and 30-degrees flexion and no features of arthrogenic muscle inhibition. MRI performed 2 days after the injury (Figure 3) showed an ACL rupture without meniscal damage.

The patient complained of anteroposterior instability and reported a preoperative Self Knee Value (SKV) score of 20. He wanted to undergo surgery with the aim of returning to competition at the Paris 2024 Paralympic Games.

**Figure 4:**
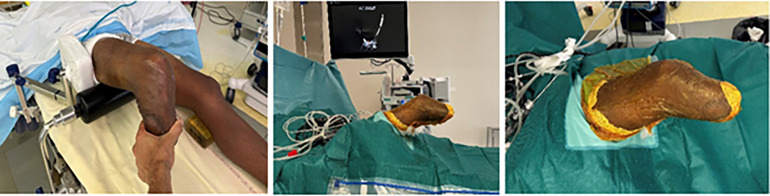
Patient installation for surgery.

The surgery was performed with the patient in the supine position under general anesthesia, with the operated leg hanging freely and a tourniquet applied at the base of the thigh. The knee was then stabilized with lateral support and a pad placed under the femur (Figure 4). This lifted up the limb and allowed full movement of the stump intraoperatively.

We carried out the ACL reconstruction using an ipsilateral quadriceps tendon graft. The quadriceps tendon was harvested through a supra-patellar incision less than 5 cm long, and the graft was subsequently woven together. Arthroscopic exploration confirmed that no meniscus damage was present. The tibial and femoral tunnels were made with outside-in instrumentation. The graft was secured to the tibia and femur using a BioComposite™ Interference Screw (Arthrex, Florida).

Lateral extra-articular tenodesis was not performed because our patient does not participate in rotational sports, only linear sports. In addition, the injury was a hyper-extension mechanism, and the clinical examination did not reveal any rotational instability. In order to avoid scar tissue and to limit conflicts with the prosthesis, we decided to limit ourselves to an isolated reconstruction of the cruciate ligament.

Post-operatively, the patient had to wear a cold splint for 3 weeks (until the knee swelling subsided) and compression stockings day and night. The patient was allowed to move the knee without restriction, and full weight bearing with a prosthesis was allowed after healing.

The follow up was provided jointly by the surgeon and sports doctors during the year following surgery.

Post-operatively, because of the reduced use of the injured side, the patient experienced a loss of muscle volume in the stump, which necessitated adjustments to his prosthesis throughout his rehabilitation by his orthoprosthetist.

The patient was able to run again after three and a half months and jump again after six months.

One year after the reconstruction, at the last follow up, the patient reported a postoperative SKV score of 95. He was able to qualify for the Paris 2024 Paralympic Games and finished 4th in his category.

## Discussion

This is the first case of ACL reconstruction in an elite amputee athlete reported in scientific literature. Previous case reports have already described ACL reconstruction in the contralateral knee, but never in the ipsilateral knee.

**Figure 5:**
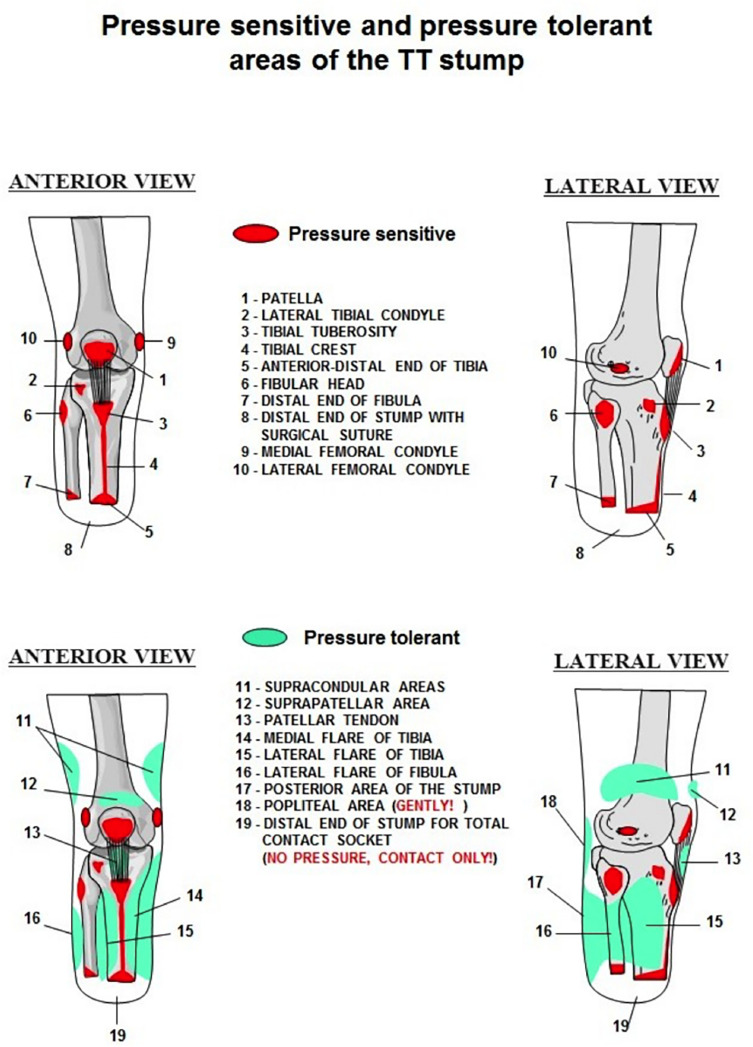
Areas of the transtibial stump that are sensitive (red) and tolerant (green) to pressure by the prosthetic [1].

The tibial prosthesis of a below-the-knee amputee consists of a socket, liner, leg segment and a foot [1, 2]. The tibial socket must distribute pressure zones to ensure comfort and provide the amputee with dynamic ability [1, 2]. It is crucial to avoid incising these sensitive areas of the stump intended for the pressure of the socket [1, 2] (Figure 5).

When selecting the graft, we did not harvest the patellar tendon graft as this structure is too close to pressure sensitive areas, including the patella.

We also did not harvest hamstring tendon as this could have reduced the athlete’s propulsive ability [3]. In a transtibial amputee, the absence of the calf muscles is compensated for by the hamstring muscles to provide propulsion [3]. Our patient is an elite athlete in long jumping, where sprinting and propulsion is key.

The running kinetics of an amputee are modified. The stride is steeper on the unaffected side because the impulse is more difficult on the side with the brace. Stance durations are 20% longer on the unaffected limb and the vertical components of the forces are 30% higher on the unaffected limb compared to the limb with the brace [3]. This information is important both from a surgical point of view, where the removal of the lateral hamstrings is not recommended, and during the rehabilitation phase to avoid compensatory overload.

When jumping, increased activation of the hamstring muscles is often found in patients after ACL reconstruction [4].

Another option would have been an allograft, which would have shortened the operation and eliminated donor-site morbidity. Nevertheless, recent studies have found an increased risk of graft failure in highly active young subjects [5].

As a result, we harvested the quadriceps tendon as we felt it was the best compromise. The incision was not in a pressure-sensitive area and did not negatively impact the patient’s ability to sprint [3] (Figure 5).

In a Paralympian athlete, the challenge was not only the surgery but also the rehabilitation. This was a truly multidisciplinary project involving surgeons, rehabilitation doctors, sports doctors, physiotherapists and orthoprosthetist. The main difference in the rehabilitation of an amputee athlete is the crucial role of the orthoprosthetist who customizes the stump socket according to the size of the stump and to each stage of rehabilitation: walking, resuming running and jumping. The second major difference is the risk of injury to the healthy limb. The main stresses are placed on the healthy limb, making it vulnerable to overuse injuries such as tendinopathies and excessive pain [3].

Rehabilitation must also be adapted to the discipline for an elite long jumper. Return to competitive sports requires not only the ability to carry out vertical jumps, tied to the concentric phase of the movement, but also horizontal jumps.

We decided to report this case because it is unique. This athletics category is extremely recent, having appeared for the first time at the Tokyo 2020 Paralympic Games. The scientific literature on the subject is therefore very limited. Longer follow-up is needed to determine whether the prosthesis will affect the graft longevity and to study the long-term implications of ACL reconstruction in this population.

## Conclusion

In a patient with a transtibial amputation, ACL reconstruction can result in excellent clinical outcomes at 1 year postoperatively, including return to high level sport. An ipsilateral quadriceps tendon graft was harvested, and the patient achieved a favorable outcome with participation in the Paris 2024 Paralympic Games.

## Data Availability

The datasets used and/or analyzed during the current study are available from the corresponding author on reasonable request.
